# The Influence of the Work of the Illinois Medical-Practice Act upon Medical Education

**Published:** 1888-12

**Authors:** H. A. Johnson

**Affiliations:** 4 Sixteenth Street


### THE INFLUENCE OF THE WORK OF THE ILLINOIS MEDICAL-PRACTICE ACT UPON MEDICAL EDUCATION.

BY H. A. JOHNSON, A. M., M. D.

Read before the American Academy of Medicine.

Among the causes that have awakened a new interest in the subject of medical education there is no one, perhaps, more important than the popular study of sanitary science. If the preservation of our homes is a question of science, the care of our bodies is also a matter of science. The American Public Health Association developed in the public mind an interest in these problems, the outcome of which has been the enactment of laws looking to a better condition, so far as sanitation is concerned, of our towns and cities.

Along with this, there soon came to be recognized a possibility of improving the instrumentalities for treatment of the sick. The agents of treatment became the subjects of legislation. Laws were passed regulating the practice of medicine. This meant that the people were satisfied that there was something more in medicine than a natural gift, something more than simply experience gained at the bedside of the patient without previous study.

The Illinois State Board of Health was created in 1877. One of the first acts of the board, as the executive of the medical-practice act, was the passage of a resolution to the effect that the diploma of a college graduating two classes in one year would not be considered to be in good standing after the first day of July, 1878. The result of this action of the board was to compel some of the prolific schools to adopt a single graduating term. It also called the attention of the profession and the people to the fact that a pressure could, in this manner, be brought to bear upon the medical colleges through legislation. If a State can exclude from practice within its own borders those who do not reach the required standard of attainments, it immediately becomes a matter of importance to the colleges that their curricula be made sufficiently broad and high to meet this standard. It was, in fact, true that persons, graduates of colleges that up to that time had full classes, were compelled to pass an examination before the board as if they were not graduates.

Some complaint was made; but there was no help for them, as the courts decided that the board was empowered to define the phrase "Medical colleges in good standing" as used in the act.

In the meantime, these schools were obliged to change their course or lose their classes. A few do still graduate two classes in the same year, but such institutions do not now graduate more than one-half of the number that annually, adorned with the parchment, formerly went forth from their doors.

In June, 1880, a committee was appointed by the board for the purpose of obtaining from the colleges themselves information upon which to base a more intelligent and comprehensive schedule of minimum requirements for practice in the State of Illinois. The board desired to know what the colleges taught, how long their lecture terms continued, the number of teachers, the conditions upon which students were admitted, and the requirements for graduation, and the proportion of graduates to matriculates. The responses to these inquiries constituted the substance of the report which was made to the board in 1883, and which, much elaborated and enriched by subsequent information, has passed into our historical literature. I have not quoted the questions but have stated the substance of them. The board adopted a schedule, which went into effect in 1884, and at the risk of wearying you I venture to include it in this paper.

I. CONDITIONS OF ADMISSION TO LECTURE COURSES.

1. Credible certificate of good moral standing. 2. Diploma of graduation from a good literary and scientific college or high school—a first grade teacher's certificate. Lacking this, a thorough examination in the branches of a good English education, including mathematics, English composition, and elementary physics and natural philosophy.

II. BRANCHES OF MEDICAL SCIENCE TO BE INCLUDED IN THE COURSE OF INSTRUCTION.

1. Anatomy. 2. Physiology. 3. Chemistry. 4. Materia medica and therapeutics. 5. Theory and practice of medicine. 6. Pathology. 7. Surgery. 8. Obstetrics and gynæcology. 9. Hygiene. 10. Medical jurisprudence.

III. LENGTH OF REGULAR GRADUATING COURSES.

1. The time occupied in the regular courses or session from which students are graduated shall not be less than five months, or twenty weeks, each. 2. Two full courses of lectures, not within one and the same year of time, shall be required for graduation with the degree of doctor of medicine.

IV. ATTENDANCE AND EXAMINATION, OR QUIZZES.

1. Regular attendance during the entire lecture course shall be required, allowance only being made for absences occasioned by the student's sickness, such absences not to exceed 20 per centum of the course. 2. Regular examinations, or quizzes, to be made by each lecturer or professor daily, or at least twice each week. 3. Final examinations on all branches, to be conducted, when practicable, by competent examiners other than the professor in each branch.

V. DISSECTIONS, CLINICS, AND HOSPITAL ATTENDANCE.

1. Each student shall have dissected during two courses. 2. Attendance during at least two terms of clinical and hospital instruction shall be required.

VI. TIME OF PROFESSIONAL STUDIES.

This shall not be less than three full years before graduation, including the time spent with a preceptor and attendance upon lectures or at clinics and hospital.

VII. INSTRUCTION.

The college must show that it has a sufficient and competent corps of instructors, and the necessary facilities for teaching, dissections, clinics, etc.

Such were the standards established, which went into effect in 1884. The graduates of schools which did not come up to these standards could not practice medicine in the State without first undergoing an examination by the board.

The statistics which have accumulated since the adoption of this schedule go to show that it has had a most important influence upon the methods and thoroughness of teaching. Previous to this time the American Medical Association and other medical societies had passed resolutions and recommendations looking to a higher standard of medical education, but there had been almost nothing accomplished outside of two or three of the leading cities. Students continued to go to those colleges which furnished them the degree with the least amount of study and money. In many instances they believed that they could make up afterward for the deficiencies of the undergraduate course, but in fact, with few exceptions, they never did.

The colleges themselves saw no reason to do what the profession did not encourage, did not even support. It is true that there were a few exceptions to this statement. In 1859 the Chicago Medical College was organized, with a graded course and a lecture term much longer than usually existed at that time. For twelve years this college was the only one in the United States with a graded course. Harvard, and subsequently the University of Pennsylvania and a few other institutions also adopted graded courses and longer terms; but at the time of the passage of the Illinois medical-practice act there had been but little advance either in the direction of a more systematic arrangement of studies or in the breadth and length of the curricula. When it was first suggested that legislation could do something to raise the standard of qualification, there were but few who believed that anything substantial could be accomplished. It was urged that the colleges were independent of legislation, and that in this free country anyone who desired to practice medicine ought to have the privilege or right to do so. The host of quacks cried "persecution," but the practical working of the law soon convinced the most skeptical that there was something being done. Many of the incompetent were driven into neighboring States, and as a result, laws similar to that of Illinois have been passed by these States in self-defense.

The last quarterly report of the secretary states that there are now 114 colleges in the United States which require evidence of some preliminary study as a condition of admission. Before the establishment of the schedule of the board, or in 1883, there were only 45. Forty-three colleges now exact attendance upon three courses of lectures as compared with 22 in 1883, and 57 others have made provision for a course of three or four years, and recommend it although they do not exact it. Hygiene is now taught in 114 colleges. In 1883 it was taught in only 42. Medical jurisprudence is now taught in 112 schools. In 1883 it was taught in 61. The lecture term has been increased, and 114 colleges now have terms of five months or more, and 61 have terms of six months or more. At the time the schedule went into effect there were 101 with five months and 42 with terms of six months.

In the meantime, institutions which have either not made any provision for instruction in some of the subjects required, or which do not exact evidence of preliminary attainments, are obliged to advertise that such of their graduates as intend to settle in Illinois may present the evidence of preliminary study and the college will certify that fact; they may also obtain instruction in the deficient branches, and this, it is thought, may save them from the humiliation of an examination by the board. As a matter of fact, it has become evident that the non-professional as well as the professional public has awakened to the importance of a more extended and a more prolonged course of study. Those colleges which make provision for this are beginning to be looked upon with more favor. They have a larger number of students of the better class, and their graduates take a higher position in the communities in which they establish themselves. All this can be verified by the facts accumulated in the reports of the board.

Of course, we do not mean to claim that all the advance of the last few years has been the direct outcome of the Illinios law. Other States have similar enactments, but it is our purpose only to speak of this State as a pioneer in this work. As we suggested at the commencement of this paper, the awakened public thought upon kindred subjects made this change a possibility. It is quite probable that the results reached would, in the absence of this public enlightenment, have been impossible a few years earlier. All that is claimed for the Illinois law is that it has been the most important factor in compelling, on the part of the colleges, a reform in their methods and means of instruction. This has been accomplished by making it impossible for the graduates of the inferior schools to practice in the State. In other words, a factory turning out inferior goods gets a bad name, and the goods do not readily find a market.

As will be seen by those who will follow the work of the board, there is a new advance to be made after the close of the session of 1890–91. At the meeting of the board in July, 1887, a resolution was adopted defining the phrase "Medical colleges in good standing" to mean "only those colleges which shall, after the sessions of 1890-91, require four years of professional study, including any time spent with a preceptor, and three regular courses of lectures, as conditions of graduation, and shall otherwise conform to the schedule of minimum requirements heretofore adopted by the board."

Information has been received that a number of schools heretofore requiring but two courses will hereafter require three courses, and there is reason to believe that there will be a very general adjustment of the breadth and length of the courses in all of the better schools so as to meet the demands of the Illinois regulations. In fact, I learn from the secretary that about one-half of the schools have already signified their intention to qualify their graduates to practice in the Prairie State.

In closing, I beg to suggest that much of the efficiency of the Illinois medical-practice act has, as it seems to me, been the outcome of a condition of things which was, when the act went into effect, a subject of criticism. I allude to the fact that the board was what is called a "mixed" body. That it was not made up of the so-called "regulars" alone, but there were among its members a homœopath, an eclectic, and two laymen. It was thought that nothing good could come out of Nazareth, and that the dignity of the profession was compromised by the association of its members with irregulars.

What was feared might be a misfortune has been in fact one principal cause of its efficiency. Instead of the irregulars increasing under the fostering care of the mixed board, they are falling off. This is, however, not the case in Illinois alone, but it is a general fact.

Upon the whole, we may well congratulate the profession of medicine in this year 1888. The numbers who gain access to our ranks are, it is true, not so numerous in proportion to the population, but they are better qualified, and there are, therefore, not so many inferior men to struggle for existence. The percentage of graduates to students is lower, which means a higher standard of attainments.

In response to a more intelligent, and, therefore, a higher estimate of the profession by the public, schools of all kinds, the so-called irregulars as well as the regulars, are spending less time upon theories and speculations, always incidental to scientific progress, and are devoting more time and effort in the endeavor to equip their graduates with a better knowledge of the established truths of medicine, a greater familiarity with the natural history of disease, and a higher skill in the use of the agents and processes by which suffering is relieved and life prolonged.

4 Sixteenth Street.

